# Quantification of GABA, glutamate and glutamine in a single measurement at 3 T using GABA‐edited MEGA‐PRESS

**DOI:** 10.1002/nbm.3847

**Published:** 2017-11-12

**Authors:** Faezeh Sanaei Nezhad, Adriana Anton, Emilia Michou, JeYoung Jung, Laura M. Parkes, Stephen R. Williams

**Affiliations:** ^1^ Centre for Imaging Science and Manchester Academic Health Sciences Centre University of Manchester Manchester UK; ^2^ Division of Neuroscience and Experimental Psychology and Manchester Academic Health Sciences Centre University of Manchester Manchester UK; ^3^ School of Medical Sciences University of Manchester Manchester UK

**Keywords:** GABA, glutamate, glutamine, MEGA‐PRESS, quantification

## Abstract

γ‐Aminobutyric acid (GABA) and glutamate (Glu), major neurotransmitters in the brain, are recycled through glutamine (Gln). All three metabolites can be measured by magnetic resonance spectroscopy *in vivo*, although GABA measurement at 3 T requires an extra editing acquisition, such as Mescher–Garwood point‐resolved spectroscopy (MEGA‐PRESS). In a GABA‐edited MEGA‐PRESS spectrum, Glu and Gln co‐edit with GABA, providing the possibility to measure all three in one acquisition. In this study, we investigated the reliability of the composite Glu + Gln (Glx) peak estimation and the possibility of Glu and Gln separation in GABA‐edited MEGA‐PRESS spectra. The data acquired *in vivo* were used to develop a quality assessment framework which identified MEGA‐PRESS spectra in which Glu and Gln could be estimated reliably. Phantoms containing Glu, Gln, GABA and N‐acetylaspartate (NAA) at different concentrations were scanned using GABA‐edited MEGA‐PRESS at 3 T. Fifty‐six sets of spectra in five brain regions were acquired from 36 healthy volunteers. Based on the Glu/Gln ratio, data were classified as either within or outside the physiological range. A peak‐by‐peak quality assessment was performed on all data to investigate whether quality metrics can discriminate between these two classes of spectra. The quality metrics were as follows: the GABA signal‐to‐noise ratio, the NAA linewidth and the Glx Cramer–Rao lower bound (CRLB). The Glu and Gln concentrations were estimated with precision across all phantoms with a linear relationship between the measured and true concentrations: R ^1^ = 0.95 for Glu and R ^1^ = 0.91 for Gln. A quality assessment framework was set based on the criteria necessary for a good GABA‐edited MEGA‐PRESS spectrum. Simultaneous criteria of NAA linewidth <8 Hz and Glx CRLB <16% were defined as optimum features for reliable Glu and Gln quantification. Glu and Gln can be reliably quantified from GABA‐edited MEGA‐PRESS acquisitions. However, this reliability should be controlled using the quality assessment methods suggested in this work.

**Abbreviations used**
ACC: anterior cingulate cortex
CC_m_: metabolite calibration coefficient
CRLB: Cramer–Rao lower bound
GABA: γ‐aminobutyric acid
Gln: glutamine
Glu: glutamate
Glx: glutamate + glutamine
HLSVD: Hankel Lanczos singular value decomposition
int_m_: metabolite intercept
LMC: left motor cortex
LOCC: left occipital cortex
MEGA‐PRESS: Mescher–Garwood point‐resolved spectroscopy
MR: magnetic resonance
MRS: magnetic resonance spectroscopy
NAA: *N*‐acetylaspartate
OCC: occipital cortex
PEPSI: proton echo planar spectroscopic imaging
PRESS: point‐resolved spectroscopy
RMC: right motor cortex
SD: standard deviation
SNR: signal‐to‐noise ratio
STEAM: stimulated echo acquisition mode

## 1 INTRODUCTION

Human brain function depends on the balance between the major inhibitory neurotransmitter γ‐aminobutyric acid (GABA) and the major excitatory neurotransmitter glutamate (Glu). GABA is a product of Glu metabolism, which is synthesized from glutamine (Gln).^1^ These three metabolites contribute to neurotransmitter cycling. Changes in each of these metabolites have been implicated in the pathophysiology of a number of neuropsychiatric and neurological disorders, such as schizophrenia,^2^ epilepsy,^3^ human immunodeficiency virus (HIV),^4^ autism^5^ and addiction.^6^ A knowledge of these metabolite levels is useful not only in understanding brain function, but also in providing biomarkers for drug discovery and treatment monitoring. Therefore, accurate and reliable estimation of these metabolites *in vivo* is of significant interest.

The chemical and structural similarities of Glu, Gln and GABA result in similar magnetic resonance (MR) spectra,^7^, ^8^ making their separation *in vivo* challenging at 3 T. Glu and Gln have been measured at 3 T using different methods, such as TE‐averaged point‐resolved spectroscopy (PRESS),^9^ short‐echo stimulated echo acquisition mode (STEAM) (TE = 6.5 ms),^10^ PRESS^11^ and proton echo planar spectroscopic imaging (PEPSI).^12^ GABA, however, has mostly been detected using *J*‐difference‐edited Mescher–Garwood point‐resolved spectroscopy (MEGA‐PRESS),^13^ which has developed into a robust and reliable method for the quantification of GABA.^14^, ^15^, ^16^, ^17^, ^18^ *J*‐difference editing is based on the quantification of the visually detectable GABA protons resonating around 3.01 ppm. The GABA C3 multiplet which is manipulated for editing resonates at 1.89 ppm close to the Glu and Gln C3 multiplets.^7^, ^8^ Thus, these amino acids co‐edit with GABA, showing peaks around 2.2–2.4 ppm and 3.75 ppm. This provides the potential to quantify Glu and Gln from the GABA‐edited acquisition.

There have been a number of studies in which Glu and Gln have been measured using GABA‐edited MEGA‐PRESS. However, reliable quantification of Glu and Gln has proven to be challenging given the low editing efficiency of Glu and Gln in this acquisition method. Some studies have quantified the peak at 3.75 ppm as Glx (the sum of Glu + Gln).^19^, ^20^, ^21^, ^22^ This approach is incapable of evaluating Glu and Gln separately and is not useful in conditions in which the Glu and Gln concentration change in opposite directions. Some studies have reported Glu concentrations using LCModel software^16^, ^23^; however, these studies fall short in quantifying Gln and cannot investigate the precision of Glu estimation.

The accuracy and precision of simultaneous measurements of Glu, Gln and GABA from edited MEGA‐PRESS spectra in phantoms at 4 T have been investigated,^24^ but translation of these results to 3 T requires further investigation and independent validation. Here, using phantoms, we first investigate the reliability of the Glx peak in GABA‐edited MEGA‐PRESS and the precision of separate Glu and Gln estimations. Then, using quality assessment of data acquired *in vivo*, we introduce a framework in order to identify the GABA‐edited MEGA‐PRESS spectra from which Glu and Gln can be estimated reliably.

## 2 METHODS

All acquisitions, *in vitro* and *in vivo*, were performed on a 3‐T MR scanner (Philips Achieva, Best, the Netherlands) using a body coil for transmission and a 32‐channel head coil for signal reception.

### 2.1 Experiments *in vitro*

Ten phantom solutions with a range of GABA, Glu and Gln concentrations and 8mM *N*‐acetylaspartate (NAA) were prepared in phosphate buffer (25mM KH_2_PO_4_, 25mM K_2_HPO_4_) and adjusted to pH 7.0 as listed in Table 1. All chemicals were from Sigma‐Aldrich (Gillingham, Dorset, UK). The phantom temperature was kept at 21°C for data acquisition. The selective pulses of MEGA‐PRESS were set to a frequency of 1.89 ppm, coupled to the detected GABA signal at 3.01 ppm, and to 7.6 ppm symmetrically disposed about the water signal. The pulses were Gaussian with a duration of 14 ms and a bandwidth of 106 Hz. TE (=TE_1_ + TE_2_) was 70 ms, with TE_1_ = 12.6 ms and TE_2_ = 57.4 ms. The spectra were acquired in blocks of four averages when the MEGA pulse was set at 1.89 ppm (MEGA‐on), referred to as a single dynamic, followed by four averages when the MEGA pulse was set at 7.6 ppm (MEGA‐off). The dynamics were then repeated in an interleaved manner until 32 were acquired at each frequency, making a total of 64 dynamics. In all scans, TR was 2000 ms with 1024 samples. The voxel size of 30 × 30 × 30 mm^3^ was chosen in the middle of the phantom. The receiver bandwidth was 2000 Hz, the water suppression method was excitation and the shimming was second‐order pencil beam (FASTMAP). The NAA peak at 2.02 ppm was used for frequency referencing.

**Table 1:** Phantom concentration

Phantom	[Glu] (mM)	[Gln] (mM)	[GABA] (mM)
1	25	0	0
2	0	25	0
3	0	0	25
4	8	2	1
5	10	4	2
6	12	6	3
7	11	2.2	2
8	9	6	2
9	15	5	3
10	18	3	3

GABA, γ‐aminobutyric acid; Gln, glutamine; Glu, glutamate.

### 2.2 Experiments *in vivo*

**Figure 1:**
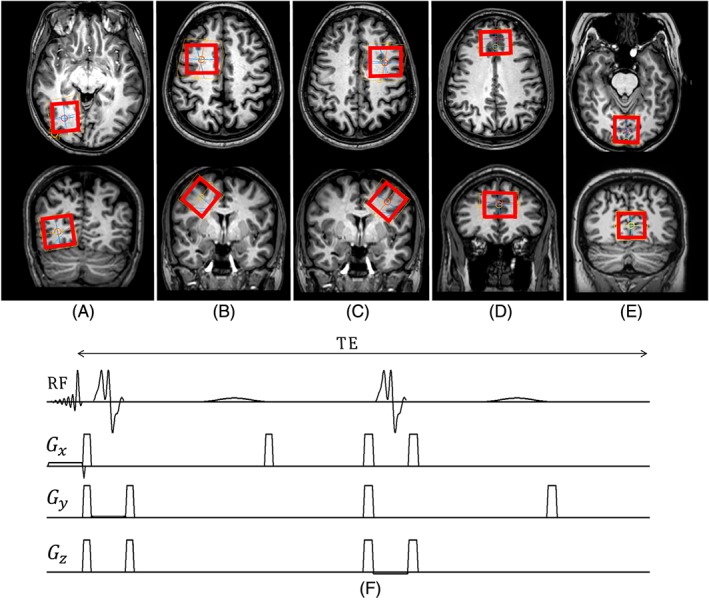
*T*_1_‐weighted images showing the positioning of the ^1^H magnetic resonance spectroscopy (MRS) voxel in the left occipital cortex (a), left motor cortex (b), right motor cortex (c), anterior cingulate cortex (d) and occipital cortex (e). (f) Diagram of the Mescher–Garwood point‐resolved spectroscopy (MEGA‐PRESS) pulse sequences used in this study

Fifty‐six spectra were acquired from 36 healthy volunteers (18 female, 18 male; age range, 20–55 years) who all gave written informed consent in accordance with the procedures approved by the local ethics committee. Ten of the volunteers had measurements in three different brain regions with a voxel size of 32 × 32 × 32 mm^3^ as follows: left occipital cortex (LOCC), left motor cortex (LMC) and right motor cortex (RMC). Seven of the volunteers had measurements in the anterior cingulate cortex (ACC), with a voxel size of 35 × 40 × 20 mm^3^, and 19 had measurements in the occipital cortex (OCC), with a voxel size of 30 × 30 × 30 mm^3^ (see Figure 1). GABA‐edited MEGA‐PRESS spectra (TE = 70 ms, TR = 2000 ms), as described in the ‘*in vitro*’ section, with identical acquisition parameters, were acquired from each voxel.

### 2.3 Metabolite quantification

Analysis of the spectroscopic data was performed in jMRUI 6.0 software^25^ using QUEST,^26^ a non‐linear, least‐squares fitting algorithm. This is a time domain algorithm which fits a weighted combination of metabolite signals directly to the data. An initial metabolite basis set for the sequence was obtained from the scanner using phantoms 1, 2 and 3 (Table 1) and a 25mM [NAA] phantom. Each phantom only contained a single metabolite. The NAA, GABA, Glu and Gln spectra from these phantoms were used as prior knowledge for QUEST quantification. For accurate frequency referencing and phase estimation of the spectrum, preprocessing was performed as follows: the dynamics which had their MEGA frequency set at 7.6 ppm (MEGA‐off spectra) were summed. The NAA peak was set as 2.02 ppm and its phase was estimated using AMARES,^27^ a resonance‐by‐resonance quantification method based on a non‐linear, least‐squares quantification. As the NAA peak in GABA‐edited MEGA‐PRESS has a 180° phase shift relative to the GABA peak at 3 ppm, the MEGA‐PRESS phase was fixed at the NAA phase minus 180°. Any residual water peak was removed using the Hankel Lanczos singular value decomposition (HLSVD) routine in jMRUI. Finally, all the dynamics were summed to give the GABA‐edited spectrum. The implementation of MEGA‐PRESS on the scanner is such that there is a phase shift of 180° between the MEGA‐on and MEGA‐off dynamics so that the GABA signal is always positive. The MEGA‐PRESS acquisition provided the possibility of a dynamic‐by‐dynamic phase and frequency correction.^28^ However, both *in vivo* and *in vitro*, the high stability of the scanner and the full cooperation of the healthy volunteers made such corrections unnecessary.

### 2.4 Concentration calculation

NAA‐referenced concentrations were estimated using the following equation:

(1) $$\left[\text{Conc}.\right]=\frac{S_{m}}{S_{\mathrm{NAA}}}\times {\mathrm{CC}}_{m}+{\mathrm{int}}_{m}$$

where *S*_m_ and *S*_NAA_ are the raw metabolite and NAA signals, respectively. In order to report accurate Glu and Gln concentrations, a calibration coefficient (CC_m_) and an intercept (int_m_) were calculated for each metabolite using the phantom data. These values were linear regression coefficients, where the *y* axis was the ratio of the metabolite (Glu or Gln) to the NAA signal intensity measured using QUEST and the *x* axis was the known concentration of the phantom (Figure 5, see later). The slope and intercept in Figure 5 (see later) are called ‘*m*’ and ‘*c*’ (conventionally *y* = *mx* + *c*), such that CC_m_ = 1/*m* and int_m_ = –*c*/*m*. These coefficients were also used to quantify the Glu and Gln concentrations *in vivo* relative to NAA, assuming a NAA concentration of 8mM (the same as in the phantoms). The calculated CC_m_ and int_m_ values were 10 and 3 for Glu and 16.6 and 1.6 for Gln.

### 2.5 Spectral classification

**Figure 2:**
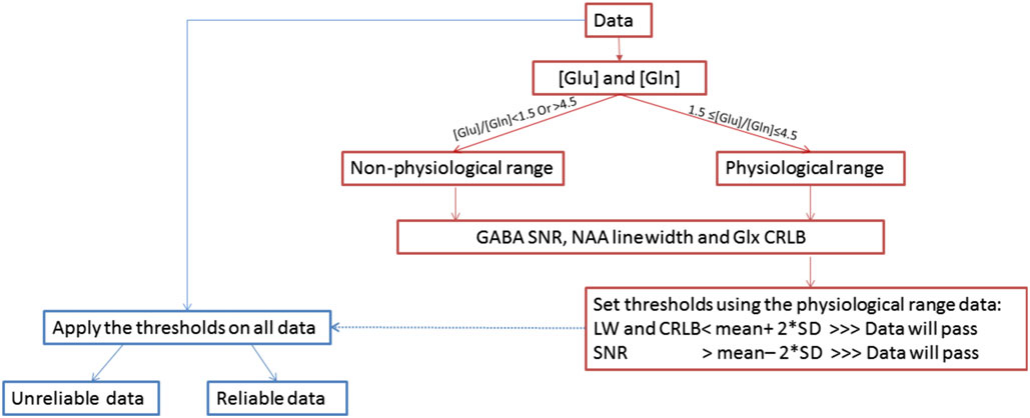
Workflow of this study. CRLB, Cramer–Rao lower bound; GABA, γ‐aminobutyric acid; Gln, glutamine; Glu, glutamate; Glx, Glu + Gln; LW, linewidth; NAA, *N*‐acetylaspartate; SD, standard deviation; SNR, signal‐to‐noise ratio

The Glu/Gln concentration ratio was used to assess the quality of the quantification for both metabolites. It was preferred over the absolute concentration as it is independent of the NAA signal and is less likely to be contaminated by errors caused by concentration calculation. The physiological concentration ranges of Glu and Gln, as reported in Govindaraju et al.,^7^ are 6–12.5mM and 3–5.8mM, respectively, leading to a ratio range of 1–4. There have been a number of studies estimating Glu and Gln by magnetic resonance spectroscopy (MRS) in different brain regions, as reviewed by Ramadan et al.^29^ In this paper, 34 studies were reported in which both Glu and Gln concentrations were measured by MRS. All studies had a ratio equal to or above 1.5; 80% of the data had a Glu/Gln ratio equal to or less than 4.5. A ratio between 1.5 and 4.5 thus encompasses most of the normal variation in Glu/Gln and is consistent with the range of absolute values reported in Govindaraju et al.^7^ This defines a range which we can be confident represents normal physiological values. This does not mean that ratios outside this range are necessarily unreliable or in error, but, in order to define a set of ‘normal’ spectra, it is prudent to exclude the values >4.5. Using this information, we defined the ratio of 1.5–4.5 as the ‘physiological range’. The spectra *in vivo* were classified into two groups: those with a Glu/Gln ratio within the physiological range and those outside this range. It is assumed that the data producing values within the physiological range are more likely to be accurate and reliable than the data producing values outside the range, and hence we investigated whether there was a difference in data quality between these two groups that can define acceptance standards. Figure 2 illustrates the flow of work in a diagram.

### 2.6 Quality assessment

**Figure 3:**
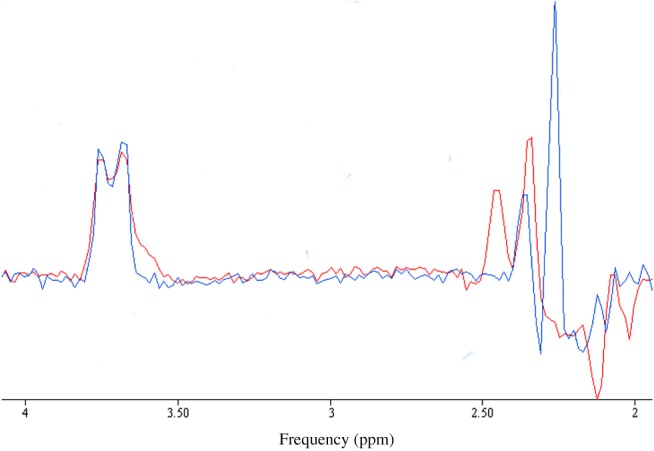
Glutamate (blue) and glutamine (red) spectra acquired from 25mM concentration phantoms

The features of a GABA‐edited MEGA‐PRESS spectrum that enable the reliable estimation of Glu and Gln concentrations are, self‐evidently, the signal‐to‐noise ratio (SNR) and spectroscopic resolution, but also the effectiveness of water suppression and scan‐to‐scan stability, as these influence the appearance of artefacts and of subtraction errors. Our approach was to estimate these independently of the QUEST analysis of the edited spectrum, and we used AMARES analysis of the spectra for this purpose. For SNR, we chose the GABA‐edited peak, as this combines the effects of intrinsic sensitivity and subtraction errors. For the linewidth, we used the NAA signal, as its proximity to the C3 region of Glu, Gln and GABA means that broad linewidths will adversely affect fitting in this region (2.2–2.4 ppm), in which the Glu and Gln overlap is minimized^23^ (see Figure 3), and is crucial to the separation of Glu and Gln. Finally, we used the Cramer–Rao lower bound^30^ (CRLB) of the fit to the edited Glx signal at 3.75 ppm. The fit to this signal is sensitive to the overall SNR, to the quality of water suppression (poor water suppression will distort the baseline in this region of the spectrum) and to subtraction errors. In the prior knowledge for AMARES, we fixed the phase of the edited signals to be 180° relative to that of NAA, so that if subtraction errors cause a phase distortion of the Glx signal, this will be reflected as a higher CRLB. AMARES performs a resonance‐by‐resonance quantification based on a non‐linear, least‐squares algorithm that requires starting values for chemical shift, linewidth and lineshape for each resonance. All peaks were quantified using defined prior knowledge, including the frequency shift and Lorentzian or Gaussian lineshape (Figure 6, see later).

To measure the GABA SNR, a single Gaussian^15^ was used to fit the GABA peak at 3.01 ppm. SNR was defined in the frequency domain as the maximum height of the fitted GABA signal at 3.01 ppm (without any line broadening) divided by the root‐mean‐square amplitude of the noise in a signal‐ and artefact‐free part of the spectrum (Figure 6, see later).

The linewidth is the full width at half‐maximum peak height in the frequency domain. The NAA linewidth was extracted from the estimation of the peak at 2.02 ppm as a Lorentzian lineshape in AMARES (Figure 6, see later).

The Glx peak at 3.75 ppm was fitted with a model extracted from the phantom data. This model was two Lorentzian lineshapes that are constrained to have identical linewidths, phases and amplitudes with a 10‐Hz separation. The CRLBs of the two Lorentzian peaks were calculated; the largest value of CRLB amongst the two was considered as the marker for quality assessment of the Glx peak (Figure 6, see later). The justification for using the larger CRLB is that any distortions or artefacts which reduce the symmetry of the two peaks will be reflected in different CRLBs for the pair of peaks.

These metrics were used to test general spectrum quality and goodness‐of‐fit. No assumptions were made that physiological range spectra were all ‘good’ or non‐physiological range spectra were ‘poor’, but rather this question was asked: do these quality metrics discriminate between these two classes of spectra? If so, this suggests that the reason for the differences in the ratio is spectroscopic quality, not an expression of biological variation. To answer this question, the mean and standard deviation (SD) of GABA‐edited SNR, NAA linewidth and Glx CRLB within each group were calculated and compared between classes.

## 3 RESULTS

### 3.1 In vitro

**Figure 4:**
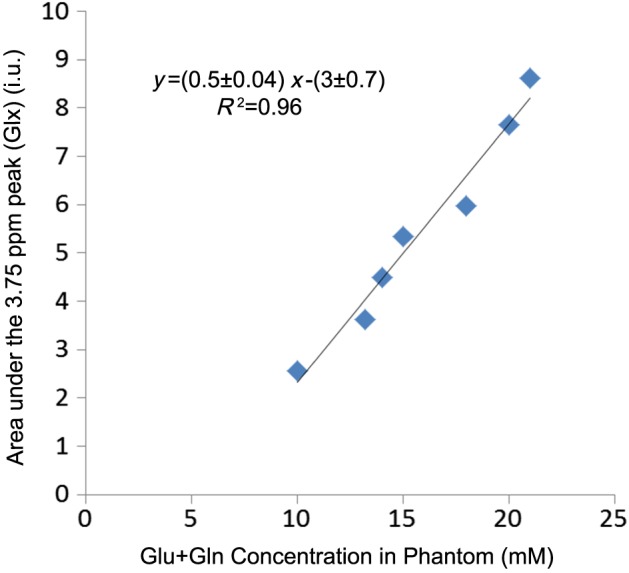
The area under the peak at 3.75 ppm, the Glx (Glu + Gln) peak, *versus* the glutamate (Glu) + glutamine (Gln) concentration present in the phantoms. The area under the peak at 3.75 ppm was quantified with AMARES

**Figure 5:**
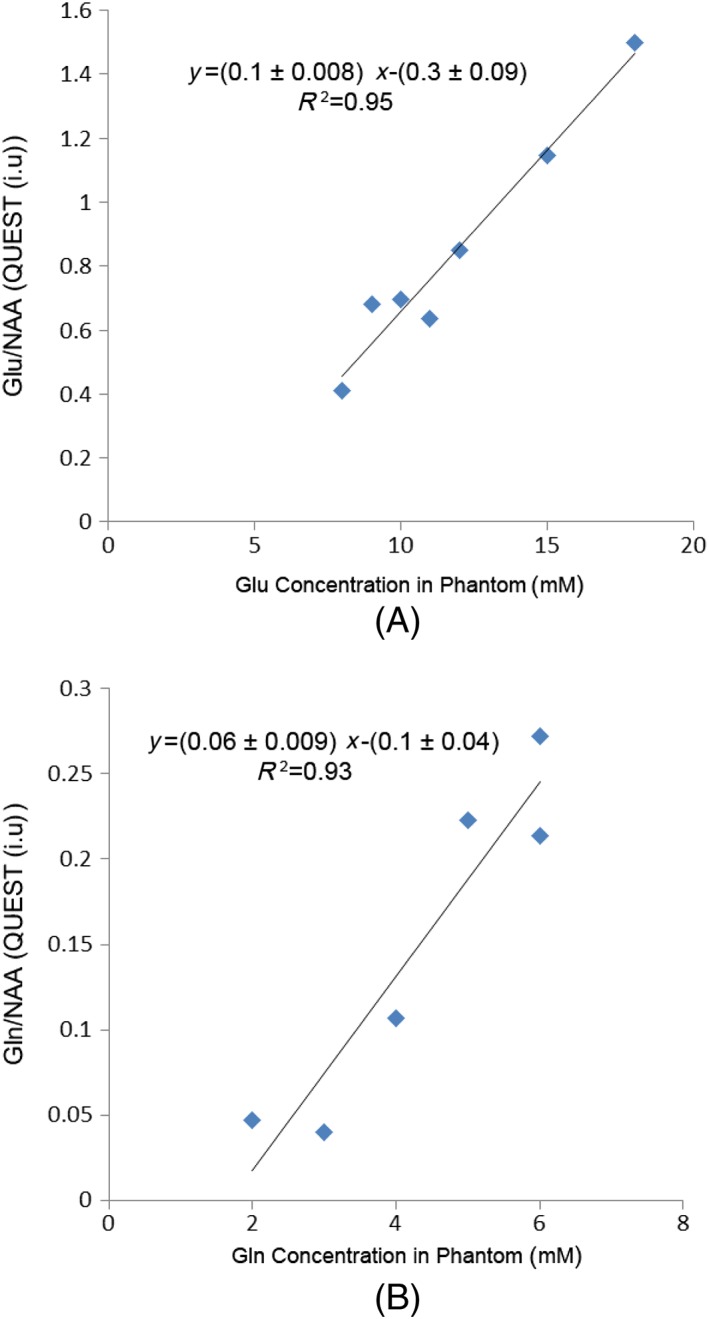
The ratio of glutamate (Glu) (a) and glutamine (Gln) (b) over *N*‐acetylaspartate (NAA) concentration quantified using QUEST in phantoms *versus* the known concentration. The slope of the line is the reciprocal of the concentration calibration coefficient (CC_m_)

The Glx peak (3.75 ppm) intensity in phantoms 4–10 (Table 1), which are a combination of Glu, Gln, GABA and NAA, with the concentration range of Glu = 8–18mM and Gln = 2–6mM, was quantified. There was a linear relationship between the peak intensity and the concentration of Glu + Gln (*R* ^1^ = 0.95, Figure 4). QUEST Glu and Gln estimation from the phantoms also had a linear relation with the known concentration. (*R* ^1^ = 0.95 for Glu and *R* ^1^ = 0.91 for Gln; Figure 5). The intercepts in the plots in Figure 5 can be interpreted as the lower concentration detection limits of this method in quantifying Glu and Gln, which are 3.51mM and 1.81mM, respectively. The calibration coefficients for converting signal ratio into concentration (CC_m_ in Equation (1)), calculated from these data, were 10 for Glu and 16.6 for Gln (Figure 5).

### 3.2 In vivo

**Figure 6:**
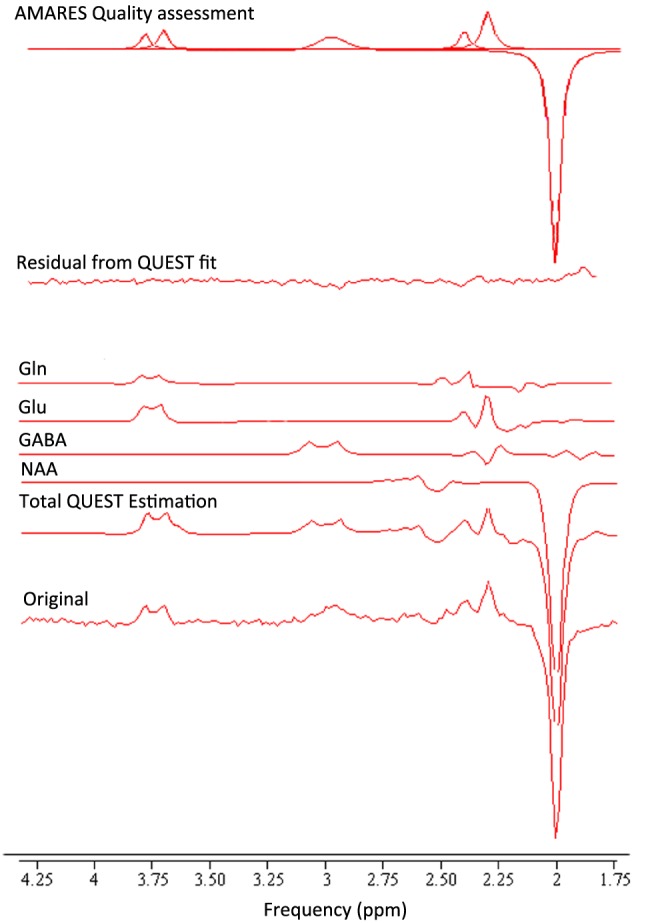
An example of a spectrum *in vivo* and its fitting. QUEST is used for concentration calculation. The individual components of the spectra glutamine (Gln), glutamate (Glu), γ‐aminobutyric acid (GABA), *N*‐acetylaspartate (NAA) and the residual are illustrated. The AMARES fit is used for quality assessment

Figure 6 illustrates the fitting of a spectrum from the data within the physiological range by both AMARES (quality assessment) and QUEST (quantification). The relatively flat residual shows that the spectrum is well described by the sum of Glu, Gln, GABA and NAA.

The number of spectra classified within the physiological range (1.5 ≤ [Glu]/[Gln] ≤ 4.5) and the mean concentration of each region are shown in Table 2. The concentrations were calculated using NAA referencing, which can introduce potential problems or bias in the data. The use of water signal referencing can reduce these putative issues, but additional water signals were not available for all *in vivo* GABA‐edited spectra.

**Table 2:** Glutamate (Glu) and glutamine (Gln) concentrations of spectra in the physiological class reported in different regions. The classification is based on Glu/Gln being equal to or between 1.5–4.5. The column ‘Number of total spectra’ includes all the spectra used in the study in each region and the column ‘Number of spectra’ includes the data classified as the physiological range. Concentration is in mmol/kg wet weight. Numbers are reported as mean ± standard deviation (SD)

	Number of total spectra	Physiological Glu/Gln ratio			
		Number of spectra	Ratio	[Glu]	[Gln]
LOCC	10	8	2.18 ± 0.25	11.07 ± 2.95	5.12 ± 1.36
LMC	10	4	3.09 ± 0.94	10.23 ± 1.24	3.51 ± 1.05
RMC	10	7	3.31 ± 0.48	13.75 ± 2.83	4.20 ± 0.98
OCC	19	18	2.53 ± 0.41	10.45 ± 0.91	4.20 ± 0.50
ACC	7	7	2.67 ± 0.62	13.55 ± 3.94	5.08 ± 0.90

ACC, anterior cingulate cortex; LMC, left motor cortex; LOCC, left occipital cortex; OCC, occipital cortex; RMC, right motor cortex.

The quality assessment markers of the physiological range class are reported in Table 3. In this class, the lower confidence limit (mean – 2SD) of the GABA‐edited SNR was 1.0, whereas the upper confidence limit (mean + 2SD) of the NAA linewidth and Glx CRLB were 8 Hz and 16%, respectively. Using these three features as quality criteria, we investigated the non‐physiological class spectra. Regardless of the fact that the classification of the spectra was independent of the quality assessment and based only on the Glu/Gln ratio, at least one of the quality criteria failed in 70% of the non‐physiological class spectra.

**Table 3:** The quality assessment markers are reported based on the classification result on the glutamate/glutamine (Glu/Gln) ratio, reported in different voxels. Numbers are reported as mean ± standard deviation (SD)

	Number of total Spectra	Physiological Glu/Gln ratio			
		Number of spectra	GABA SNR	NAA LW	Glx CRLB
LOCC	10	8	2.63 ± 0.37	6.20 ± 0.68	6.97 ± 1.89
LMC	10	4	2.22 ± 0.43	6.04 ± 0.75	14.19 ± 8.02
RMC	10	7	2.22 ± 0.84	6.19 ± 0.97	9.04 ± 6.51
OCC	19	18	2.41 ± 0.48	6.17 ± 0.96	5.02 ± 1.21
ACC	7	7	3.97 ± 1.02	5.61 ± 0.45	6.27 ± 4.23

ACC, anterior cingulate cortex; CRLB, Cramer–Rao lower bound; GABA, γ‐aminobutyric acid; Glx, Glu + Gln; LMC, left motor cortex; LOCC, left occipital cortex; LW, linewidth; NAA, *N*‐acetylaspartate; OCC, occipital cortex; RMC, right motor cortex; SNR, signal‐to‐noise ratio

**Figure 7:**
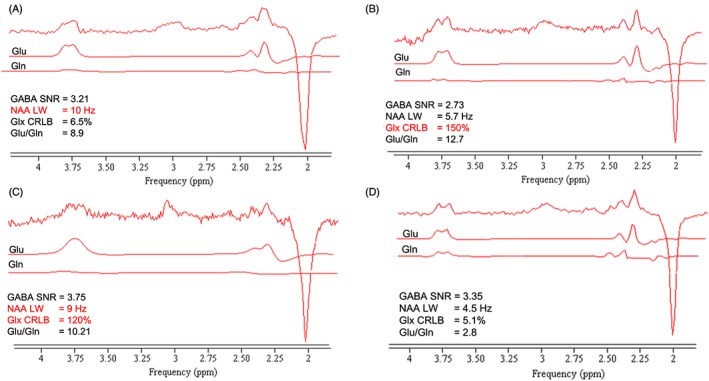
(a–c) Examples of glutamate (Glu) and glutamine (Gln) fitting in spectra *in vivo* which failed one or two checks in the quality assessment. The upper trace is the original data and the QUEST fits of Glu and Gln are presented below. The quality assessment values calculated using AMARES are reported for each spectrum; the text in red indicates the failed quality parameter. (d) Sample of a spectrum and its Glu and Gln fit which passed all the quality criteria checks. CRLB, Cramer–Rao lower bound; GABA, γ‐aminobutyric acid; Glx, Glu + Gln; LW, linewidth; NAA, *N*‐acetylaspartate; SNR, signal‐to‐noise ratio

Investigation of the failed spectra showed that the most common feature that fell short in the criteria was CRLB, with eight of 56 spectra not meeting the requirement, and the NAA linewidth, with four of 56 falling short. The SNR criteria did not fail any spectra, and so this does not form part of the quality assessment framework. Overall, the proportion of spectra which failed the framework in this dataset was region dependent, with the greatest in the motor cortex (8/20) and the least in OCC (3/29). Figure 7 shows examples of spectra in which the quality threshold failed on a single feature (Figure 7a, b), two features (Figure 7c) and a spectrum which passed all quality thresholds (Figure 7d).

**Figure 8:**
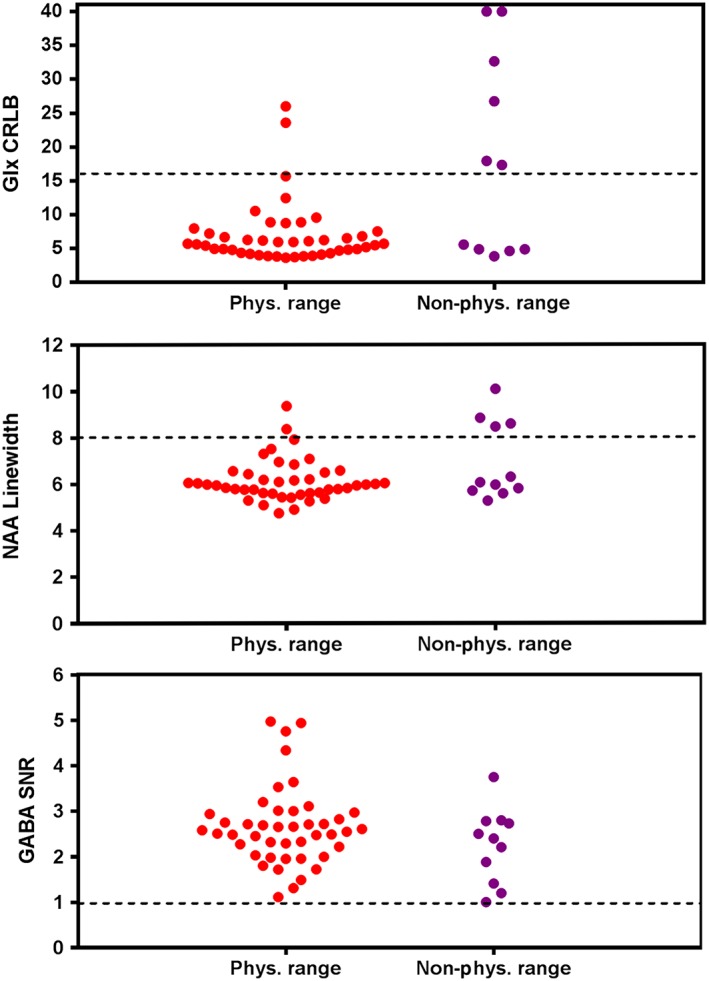
Scatter plots of the three quality assessments: Glx (glutamate + glutamine) Cramer–Rao lower bound (CRLB), *N*‐acetylaspartate (NAA) linewidth and γ‐aminobutyric acid (GABA) signal‐to‐noise ratio (SNR) are illustrated in both the physiological (Phys.) and non‐physiological (Non‐phys.) classes of data. The broken line is the rejection threshold

**Figure 9:**
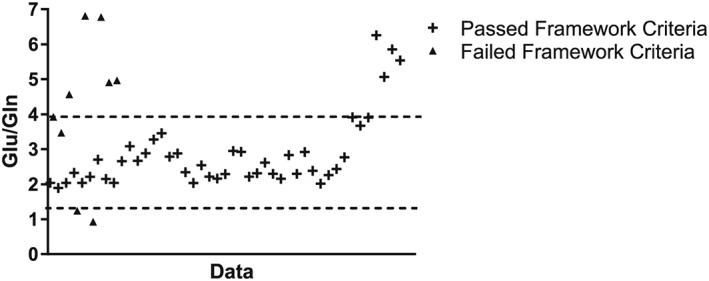
Plot of all the data points and their ratio of glutamate to glutamine (Glu/Gln). The triangles represent the data that failed the quality criteria and the crosses are those that passed. The broken line indicates the Glu/Gln ratios of 1.5 and 4.5

Figure 8 shows a scatter plot of the quality assessment criteria in both physiological and non‐physiological groups. Figure 9 shows a plot of all the data points and their ratio of [Glu]/[Gln]. It can be seen that there are data points which fail the quality assessment framework whilst being in the physiological range class, and vice versa (data points that pass the framework and do not belong to the physiological range class). Investigation of the data also showed that the majority of the failed spectra underestimate the Gln concentration, resulting in a high Glu/Gln ratio.

## 4 DISCUSSION AND CONCLUSIONS

The linear correlation of the Glx peak with the total Glu + Gln present in the phantoms shows that the peak at 3.75 ppm can be used as an indicator of Glu + Gln, but cannot be used as a sole marker for Glu. The linear correlation of the Glu and Gln estimation with the concentration present in the phantom shows that, in ideal conditions, Glu and Gln can be independently quantified with precision using GABA‐edited MEGA‐PRESS and QUEST. This provides the potential to quantify Glu, Gln and GABA from a single acquisition at 3 T.

The calibration coefficient (CC_m_) for Glu and Gln not only reflects the relationship between the signal intensity and concentration, but also the editing efficiency. Having a smaller value of CC_m_ for Glu (10) compared with Gln (16.6) suggests that this metabolite is more effectively edited in GABA‐edited MEGA‐PRESS. This is to be expected as the C3 multiplet of Glu (at 2.03 ppm) is 0.07 ppm closer to the editing pulse (at 1.89 ppm) than that of Gln (at 2.1 ppm).^7^

The application of the quality assessment framework to the data suggests that there is a difference in spectroscopic quality between regions. More than 90% of the data in OCC, LOCC and ACC passed the quality assessment, whereas, in the motor cortex (LMC, RMC), only 60% of the data did so. This regional dependence can be explained as a result of the difficulty of performing spectroscopy in different brain areas. The motor cortex is relatively harder to shim, and is more susceptible to outer‐volume lipid contamination. The location of the voxel directly determines the quality of the spectra and hence can be a factor in estimating the possibility of Glu and Gln quantification in GABA‐edited MEGA‐PRESS.

Glu and Gln quantification from an acquisition that is not optimized for these metabolites is complex, and reliable estimation of these metabolites requires extra quality measurements. Figure 8 shows that none of the three markers (GABA‐edited SNR, NAA linewidth or Glx CRLB) on their own can identify the spectra with unreliable Glu and Gln estimation. However, using the combination of NAA linewidth and Glx CRLB can resolve this problem.

The classification of reliable Glu/Gln estimation based on their ratio is a method to compensate for the lack of knowledge of the true concentrations present *in vivo*. This does not imply that the Glu/Gln ratios outside the defined value are unacceptable. A number of studies in Ramadan et al.^29^ reported a Glu/Gln ratio higher than 4.5. In addition, in our dataset, three data points pass the quality assessment framework regardless of having a Glu/Gln ratio higher than 4.5. Here, we used the most reliable data to develop a framework in order to identify the features of good‐quality spectra. However, in our dataset, seven of 11 of the estimations which did not have a Glu/Gln ratio between 1.5 and 4.5 failed in one or both of the quality assessment criteria. Although it is inevitable that 95% of the spectra within the ratio limits will meet the quality criteria (as this is how the criteria were defined), this does not mean that all the spectra outside these limits have to fail. The fact that the majority of the data do fail in at least one feature gives some confidence that the quality thresholds are useful. The use of the quality assessment step may result in the rejection of a proportion of the data, such that the statistical power may be reduced, and this needs to be taken into account in study design. Care should be taken to ensure that, if spectra are rejected, this occurs in a balanced manner across experimental groups, as a preponderance of failed spectra in one group could bias the overall results.

In conclusion, Glu and Gln can be quantified reliably from GABA‐edited MEGA‐PRESS under certain circumstances. Quality assessment of the spectra and the framework proposed in this study can be used as a tool to evaluate the reliability of the Glu and Gln quantification from GABA‐edited MEGA‐PRESS spectra.
